# Iron, Ferroptosis, and Head and Neck Cancer

**DOI:** 10.3390/ijms242015127

**Published:** 2023-10-12

**Authors:** Yong Teng, Lixia Gao, Antti A. Mäkitie, Ewa Florek, Agata Czarnywojtek, Nabil F. Saba, Alfio Ferlito

**Affiliations:** 1 Department of Hematology and Medical Oncology, Winship Cancer Institute, Emory University School of Medicine, Atlanta, GA 30322, USA; nfsaba@emory.edu; 2 Wallace H. Coulter Department of Biomedical Engineering, Georgia Institute of Technology and Emory University, Atlanta, GA 30322, USA; 3 College of Pharmaceutical Sciences and Chinese Medicine, Southwest University, Chongqing 400715, China; lx126001@126.com; 4 Department of Otorhinolaryngology-Head and Neck Surgery, Research Program in Systems Oncology, Faculty of Medicine, University of Helsinki and Helsinki University Hospital, FI-00014 Helsinki, Finland; antti.makitie@helsinki.fi; 5 Laboratory of Environmental Research, Department of Toxicology, Poznan University of Medical Sciences, 60-631 Poznan, Poland; eflorek@ump.edu.pl; 6 Department of Pharmacology, Poznan University of Medical Sciences, 60-806 Poznan, Poland; agata.czarnywojtek@ump.edu.pl; 7 Department of Endocrinology, Metabolism and Internal Medicine, Poznan University of Medical Sciences, Przybyszewskiego 49, 60-355 Poznan, Poland; 8 Coordinator of the International Head and Neck Scientific Group, 35125 Padua, Italy; profalfioferlito@gmail.com

**Keywords:** ferroptosis, head and neck cancer, iron, cell death, antitumor strategy

## Abstract

Ferroptosis is an iron-dependent regulatory form of cell death characterized by the accumulation of intracellular reactive oxygen species and lipid peroxidation. It plays a critical role not only in promoting drug resistance in tumors, but also in shaping therapeutic approaches for various malignancies. This review aims to elucidate the relationship between ferroptosis and head and neck cancer treatment by discussing its conceptual framework, mechanism of action, functional aspects, and implications for tumor therapy. In addition, this review consolidates strategies aimed at improving the efficacy of head and neck cancer treatment through modulation of ferroptosis, herein serving as a valuable reference for advancing the treatment landscape for this patient population.

## 1. Introduction

**Figure 1:**
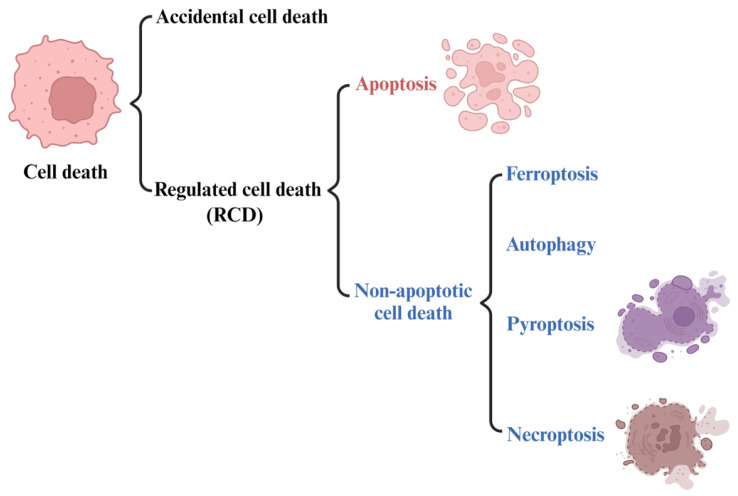
The types of cell death. Regulated non-apoptotic forms of cell death include ferroptosis, autophagy, pyroptosis, and necroptosis.

Iron is an essential trace element in mammals, which plays an important role in DNA synthesis, electron transport, oxygen transport, and other metabolic processes [1,2]. Under normal conditions, cell iron metabolism is in a dynamic balance of constant absorption, utilization, storage, and circulation. This process is called iron balance and plays an important role in maintaining the normal physiological function of cells. However, when the iron balance in cells is disrupted, it can lead to intracellular iron overload. Excess iron produces reactive oxygen species (ROS) through the Fenton reaction [3], which can induce ferroptosis, which is different from other forms of cell death, such as apoptosis, paraptosis, and necrosis (Figure 1) [4]. ROS is mainly produced by iron-dependent Fenton reactions, mitochondria, or enzymes from the nicotinamide adenine dinucleotide phosphate oxidase (NADPH) family [5]. Because iron is an essential element for all cell growth, the rapid proliferation of tumor cells is usually more dependent on iron than normal cells are. As a result, tumor cells are more sensitive to the damage associated with iron excess [6].

Ferroptosis is a newly defined form of iron-dependent cell death with excessive accumulation of lipid peroxidation [7,8]. Due to their potential for damaging biofilms and trigger a cascade of lipid peroxidation, excess ROS play a significant role in various forms of cell death, including ferroptosis and autophagy [9]. Some common tumor chemotherapy drugs, such as sorafenib, 5-FU, and paclitaxel, can cause cytotoxicity by triggering ROS accumulation and lipid peroxidation to kill tumor cells. Accumulation of iron ions has been demonstrated in various tumor cells [10,11,12]. Therefore, regulating ferroptosis through iron ion homeostasis provides a novel strategy for killing tumor cells [13].

Ferroptosis is not only related to the occurrence and development of many diseases; key proteins in the related signaling pathway can also become drug targets. In recent years, studies on ferroptosis and cancer have increasingly emerged, focused on tumor cell death, tumor metastasis, tumor immunity, tumor drug resistance, and tumor therapy [14,15,16,17]. One study has comprehensively evaluated the role of ferroptosis versus iron-growth-related genes (FRGs) in the prognostic prediction and immunotherapy of glioma patients through bioinformatics, and the involvement of ferroptosis in the immunotherapy of glioma is expected to become a new method for predicting the immunotherapy of glioma [18]. Activation of epithelial mesenchymal transformation (EMT) is pivotal in driving both tumor cell metastasis and drug resistance. Studies have shown that EMT cells are highly sensitive to ferroptosis [19,20,21]. Both radiotherapy and chemotherapy used in cancer treatment can cause ferroptosis. In conclusion, ferroptosis plays an important role in tumor treatment. 

## 2. Ferroptosis

### 2.1. The Development of the Concept of Ferroptosis

For a long time, cell death has been divided into three forms based on morphological characteristics: apoptosis, autophagy, and necrosis [22]. With the advancement in research, new types of cell death are emerging, and each type shows different characteristics in terms of molecular mechanisms and regulatory signals. Ferroptosis was originally discovered by targeting drugs against RAS mutations. RAS is the first identified and the most common human oncogene [23]. It is the most conserved family of oncogenes known to date and plays an important role in cell growth, proliferation, differentiation, regulation, and malignant transformation [24,25]. Ferroptosis was originally identified and named in cancer cell experiments with RAS mutations, and ferroptosis agonists are capable of killing such tumor cells in vitro [26]. Two small molecule compounds (erastin and RSL3) were initially found to have specific killing effects on cancer cells expressing oncogenic RAS compared with wild-type cells [27]. This study further found that erastin- or RSL3-induced cell death differs from traditional apoptosis in that it is not blocked by the pan-caspase inhibitor (Z-VAD-FMK) but can be blocked by iron-chelating agents. Based on the above findings, ferroptosis was preliminarily described as an iron-dependent form of non-apoptotic cell death.

In 2018, the cell death committee made an updated recommendation on cell death from morphological, biochemical, and functional perspectives [28]. The types of cell death can be classified into accidental cell death (ACD) and regulated cell death (RCD) (Figure 1). RCD is a homeostasis mechanism in multicellular organisms, which is essential for maintaining the morphology and function of cells and can be regulated at the genetic and pharmacological levels [29]. The RCD category at least includes five high-profile forms, including apoptosis, pyroptosis, autophagy, necroptosis and ferroptosis (Figure 1). However, ferroptosis, as a newly identified cell death mechanism, is attracting increasing attention. The concept of ferroptosis was first proposed by Dr. Brent R Stock in 2012 [27,30,31] as a form of iron-dependent regulatory cell death that occurs through the accumulation of toxic lipid ROS and the consumption of polyunsaturated fatty acids, resulting in mechanical damage to cells. It also can be defined as being triggered by oxidative perturbation of the intracellular microenvironment, controlled by glutathione peroxidase 4 (GPX4), which can be inhibited by iron-chelating agents and lipophilic antioxidants [32]. In other words, ferroptosis is caused by a decrease in the activity of a key enzyme responsible for repairing oxidative damage to cell membranes, GPX4 [33]. GPX4 protects cells by removing harmful substances from iron-dependent lipid peroxidation and causes ferroptosis when it malfunctions. 

### 2.2. Features and Components of Ferroptosis

Programmed cell death is an active and orderly cell death determined by genes and regulated by molecular mechanisms. It is closely related to the maintenance of homeostasis and the occurrence of diseases, and mainly includes pyroptosis, necrosis, and autophagy. As a newly discovered form of programmed cell death, ferroptosis is characterized by iron-dependent lipid peroxidation [34]. The typical morphological features of ferroptosis are reduced mitochondrial volume, increased double membrane density, reduced or disappeared mitochondrial ridge, broken mitochondrial outer membrane, normal nuclear size, and lack of chromatin aggregation. The biochemical characteristics of ferroptosis are different from those of apoptosis, autophagy, and necrosis (Table 1). When the metabolism of the cellular antioxidant system is abnormal, Fe^2+^ accumulation can mediate the Fenton reaction to produce excess ROS (especially hydroxyl free radicals). The peroxidation of ROS with polyunsaturated fatty acid (PUFA) on the cell membrane causes the stability of the lipid bilayer to be destroyed and the cell membrane to disintegrate, which in turn induces cell ferroptosis [27].

Lipid peroxidation and ROS are two significant markers of ferroptosis. Lipid peroxidation, which often leads to lipid hydroperoxide formation, occurs in response to oxidative stress. Phospholipid peroxidation represents a critical stage within the ferroptosis process, which is closely associated with a variety of human diseases. In addition, the process of ferroptosis is accompanied by changes in related genes and proteins, such as ACSL4, TP53, GPX4, HSPB1, ACSL4, TFRC, ACSF2, and others (Table 2) [35,36,37,38,39,40,41,42,43,44,45,46,47,48,49,50]. In recent years, new genes and proteins involved in ferroptosis have also been discovered.

### 2.3. The Mechanism and Regulation of Ferroptosis

**Figure 2:**
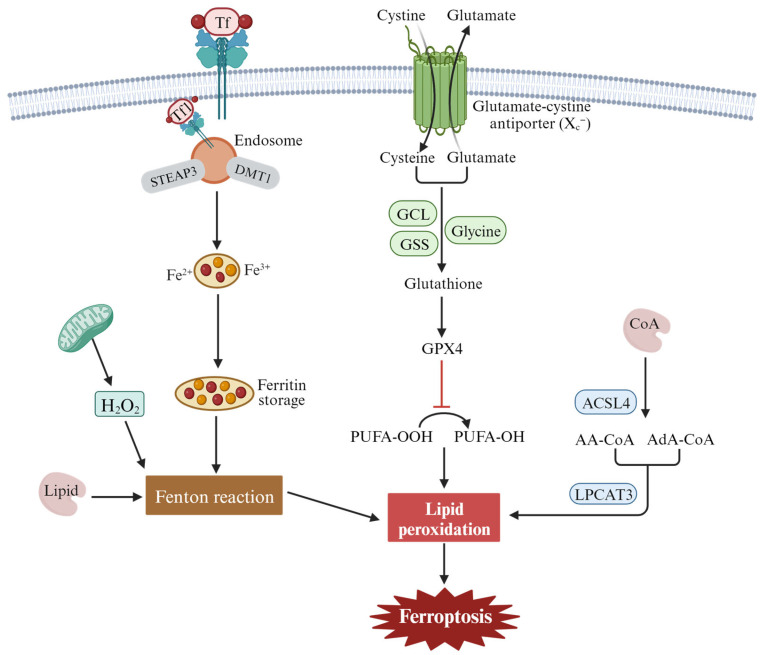
The main regulatory mechanisms of ferroptosis. These mechanisms include the inhibition of the cystine/glutamate antiporter system (system x_c_^−^), leading to depletion of intracellular glutathione and the accumulation of lipid peroxidation products due to the oxidation of polyunsaturated fatty acids (PUFAs) by reactive oxygen species (ROS) and the dysregulation of iron metabolism through transferrin receptor 1 (Tf1).

Ferroptosis can be divided into extrinsic and intrinsic pathways. The extrinsic pathway refers mainly to transporter-dependent pathways, while the intrinsic pathway refers to pathways regulated by various enzymes [35,51,52,53] (Figure 2). Extrinsic pathways are initiated by the inhibition of cell membrane transporters such as cystine/glutamate reverse transporters (System x_c_^−^) or by the activation of the iron transporters’ serum transferrin and lactotransferrin. System x_c_^−^ exchanges intracellular glutamate for extracellular cystine (Cys2), which is catalyzed by glutamate-cysteine ligase (GCL) and glutathione synthetase (GSS) to synthesize glutathione (GSH). Inhibiting the activity of System xc- can inhibit the absorption of cystine, affect the synthesis of GSH, lead to a decrease in activity of the membrane lipid repair enzyme GPX4, reduce the antioxidant capacity of cells, and eventually induce ferroptosis of cells. In addition, increased iron accumulation through increased iron absorption, reduced iron storage, and limited iron outflow can also promote ferroptosis. Under pathological conditions, Fe^2+^ accumulates in the cell and produces a large number of ROS, which occurs in the Haber–Weiss and Fenton reactions. A series of peroxidation reactions occurs with the polyunsaturated fatty acid PUFA on the cell membrane to generate lipid peroxides, which destroy the cell membrane structure and cause ferroptosis in the cell. Transferrin (serum transferrin or lactoferrin, Tf) mediates iron uptake through the transferrin receptor (TFRC), and FTH1/FTL (ferritin components) increases iron levels through autophagy degradation, all of which can contribute to ferroptosis.

The intrinsic pathway of ferroptosis is activated by enzymes, mainly through lipid metabolism and other metabolism [53]. The central mechanism in ferroptosis is the iron-dependent lipid oxidation metabolism disorder, and PUFAs are the key substances of lipid peroxide accumulation in ferroptosis. Under normal circumstances, PUFAs are important substrates for lipid metabolism, containing diallyl hydrogen atoms, especially arachidonoyl (AA) and renal/supra-adenoid/adrenoyl (AdA), which easily react with ROS and cause lipid peroxidation. Long-chain fatty acyl-CoA synthetase 4 (ACSL4) catalyzes free AA or AdA to combine with coenzyme A (CoA) to form the derivative AA-CoA or ADA-CoA, which is then esterified into membrane phosphatidyl ethyl/alcohol/amine (PEs) by lysophosphatidyllecithin acyltransferase 3(LPCAT3). After oxidation through lipoxygenase (ALOXs) or cytochrome P450 oxidoreductase (POR), harmful lipid peroxidation products are formed, which induce cell ferroptosis. 

Fatty acid synthesis mediated by acetyl-CoA carboxylase (ACAC) and fatty acid release mediated by lipophagy can induce the accumulation of free fatty acids in cells and promote ferroptosis [54,55]. p53 can induce ferroptosis by downregulating the expression of the system x_c_^−^ component SLC7A11 and inhibiting cystine uptake. At the same time, p53 can inhibit the activity of dipeptidyl peptidase-4 (DPP4) and block erastin-induced ferroptosis [56]. Nrf2 is an important regulatory factor in the maintenance of intracellular REDOX homeostasis. Through the p62-Keap1-Nrf2 pathway, the expression of multiple genes involved in iron and ROS metabolism (NQO1, HO1, and FTH1) is upregulated, and cell ferroptosis is inhibited [57]. GPX4 reduces cytotoxic lipid peroxides (L-OOH) to the corresponding alcohols (L-OH), and inhibition of GPX4 activity leads to the accumulation of lipid peroxides in cell membranes. For example, RSL3, as an inducer of ferroptosis, can directly act on GPX4 and inhibit its activity, thus reducing the antioxidant capacity of cells and accumulating ROS, resulting in ferroptosis [27]. However, this research is now controversial, with newer studies finding that RSL3 and ML162 do not have the ability to inhibit the enzyme activity of the recombinant selenium protein GPX4. Surprisingly, another selenium protein, TXNRD1, can effectively inhibit GPX4 activity [58]. Therefore, the search for direct inhibitors of GPX4 needs to continue. According to the above summary, the proteins involved in ferroptosis are mainly GPX4, FSP1, p53, Nrf2, ATF4, P62, and others. In addition, some small-molecule compounds, drugs, or certain stresses can also induce ferroptosis.

## 3. The Function of Ferroptosis in Head and Neck Squamous Cell Carcinoma (HNSCC)

HNSCC is the sixth most common cancer in humans worldwide and is associated with a poor prognosis for patients [59]. Although there have been continuous advances in the field of surgery, radiotherapy, and chemotherapy in recent years, the 5-year survival rate of HNSCC has not significantly improved, and 30–40% of patients are likely to develop distant metastasis within 5 years [60,61,62]. To improve the diagnosis and treatment of HNSCC, it is important to understand the cellular mechanisms of HNSCC development and metastasis. The antitumor effect of ferroptosis in HNSCC has been extensively investigated (Table 2). Here, we mainly discuss the role of ferroptosis in the onset and progression of HNSCC, as well as its potential in treatment. This exploration aims to establish a foundation for enhancing therapeutic approaches for HNSCC.

### 3.1. Ferroptosis and Tumor Cell Death

Interferon γ (IFN-γ) has been implicated in T helper type 1 (Th1) cell development through its ability to optimize interleukin 12 (IL-12) production from macrophages and IL-12 receptor expression on activated T cells. As a new mode of action for cytotoxic T-cell-mediated tumor killing, T-cell-derived IFN-γ stimulates ACSL4 and alters tumor cell lipid patterns by binding to arachidonic acid to induce ferroptosis in immunogenic tumor cells [63]. In a clinical context, the presence of tumor ACSL4 is associated with T cell markers and improved survival in cancer patients treated with immune checkpoint blockade (ICB) therapy. Therefore, the combination of IFN-γ signaling and specific fatty acids, such as arachidonic acid, represents an inherent mechanism for promoting ferroptosis within tumors and underscores the role of cytotoxic T cells in this process. Studies have revealed that erlotinib can promote the production of ROS in HNSCC cells and induce ferroptosis by inhibiting EGFR, thus killing tumor cells [64]. Dihydroartemisinin (DHA), a semi-synthetic derivative of artemisinin, has high antitumor biological activity. DHA was used to treat five HNSCC cell lines and two non-oncogenic normal epithelial cell lines. The results showed that DHA induces cell cycle arrest through Forkhead box protein M1 (FOXM1) and induces ferroptosis and apoptosis in HNSCC. Therefore, DHA may be promising for the treatment of HNSCC [45]. It has been reported that ferroptosis can affect the efficacy of chemotherapy, radiotherapy, and immunotherapy, so the killing of tumor cells by drugs targeting ferroptosis signals may be a potential new strategy to improve the antitumor therapeutic effect. It was found that ferrostatin-1, as a common ferroptosis inhibitor, could effectively inhibit the production of HPEPEt-PE by the 15LOX/PEBP1 complex, but had no effect on 15LOX [65]. A study has shown that PEBP1, a scaffold protein inhibitor of protein kinase, can bind to 15LO to affect the production of hydroperoxy-PE and significantly contribute to disease development that is dependent on iron death, suggesting that the PEBP1/15LO complex has potential value for application in drug discovery [66]. Further research found that redox reprogramming of 15-lipoxygenase (15-LOX) can produce pro-ferroptotic signals, 15-hydroperoxy-eicosa-tetraenoyl-phosphatidylethanolamine (15-hPEt-PE), which modulate the ferroptotic endurance. The main mechanism is iNOS, which is involved in regulating the arachidonoyl—phosphatidylethanolamines (ETE—PE) REDOX state under the condition of proinflammatory-induced ferroptosis [67].

### 3.2. Ferroptosis and Tumor Metastasis

Tumor metastasis is the main factor in malignant tumor treatment failure. The importance of ferroptosis in tumor metastasis has attracted increasing attention. It was found that the ferroptosis driver SOCS1 and inhibitor FTH1 are closely related to the degree of macrophage infiltration in HNSCC, and both can be used as prognostic indicators for HNSCC, suggesting that ferroptosis plays an important role in the infiltration process of HNSCC [43]. The study showed that low expression of the DNA-damage-inducible transcript 4 (DDIT4) could significantly inhibit the invasion and migration of HNSCC cells, but overexpression of DDIT4 was negatively correlated with cell infiltration. It was suggested that DDIT4 plays a key role in HNSCC metastasis and may be a potential target for HNSCC treatment [68].

### 3.3. Ferroptosis and Antitumor Immunity

**Figure 3:**
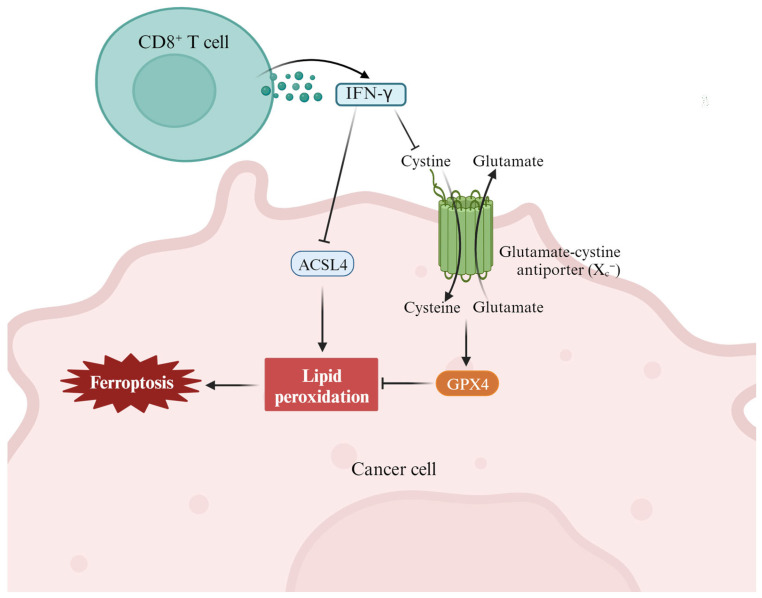
The cross-talk between the immune system (such as CD8^+^ T cells and IFN-γ) and lipid metabolism (such as different fatty acids) in the context of tumor ferroptosis in the tumor microenvironment. In this case, T-cell-derived IFN-γ stimulates ACSL4 and alters tumor cell lipid patterns by binding to arachidonic acid to induce ferroptosis in immunogenic tumor cells [63].

Immunotherapy is one of the most promising antitumor therapies and is achieved by activating the immune system to enhance its inherent cancer treatment ability [69]. In recent years, ferroptosis has been found to be closely related to tumor immunotherapy (Figure 3) [70,71,72]. With the development of immunology research, the combination of immunotherapy and ferroptosis will provide a new therapeutic strategy for HNSCC patients. Yang et al. found that ferroptotic stress can induce PD-L1 expression in HNSCC by regulating the NF-KB signaling pathway and calcium influx through ROS [37]. This study reveals a subgroup of ferroptotic HNSCC with immune-active signatures and indicates the potential for ferroptosis inducers to increase the HNSCC efficacy of immune checkpoint inhibitors. Zhang et al. showed that PKCβII senses initial lipid peroxides and activates ferroptosis-related lipid peroxidation through phosphorylation of ACSL4 [73]. Studies have also shown that attenuation of the PKCβII-ACSL4 pathway effectively blocks ferroptosis in vitro and impairs ferroptosis-related cancer immunotherapy in vivo. Gao et al. found that the combination of IFN-γ released by CD8^+^ T cells and arachidonic acid (AA) induces the apoptosis of iron in immunogenic tumors [63]. The specific mechanism is that IFN-γ stimulates ACSL4 and changes the lipid pattern of tumor cells, thereby increasing the binding of AA with C16 and C18 acyl-chain phospholipids. C16 and C18 can promote ACSL4-dependent tumor ferroptosis induced by IFN-γ and AA. According to the above two studies, targeting the PKCβII-ACSL4 signaling pathway can be used as a new strategy for inducing tumor ferroptosis and immunotherapy [74].

A study using bioinformatics techniques obtained 21 important ferroptosis- and pyroptosis-related genes (FPRGs) based on a training dataset (TCGA-HNSC) via univariate Cox and differential expression analysis, combined with a line graph model of risk score and clinical information. Finally, nine FPRGs were selected as molecular markers and effective immunotherapy targets for HNSCC treatment [75]. Rhythmic mild-temperature photothermal therapy (mPTT) based on organic photothermal nanoparticles (PBDBT NPs) has been shown to kill oral squamous cell cells and induce the death of immunogenic cancer cells in vitro and in vivo, showing strong efficacy of mPTT and active tumor-specific adaptive immune responses [76]. Unfortunately, the authors did not test the 21 important FPRGS in this study. The researchers suggested that mPTT may have the potential for clinical application. In HNSCC tissue cohorts, the expression of GPX4 was negatively correlated with the immunogenic cell-death-related protein calreticulin. The GPX4 inhibitor RSL3 could induce ferroptosis in nasopharyngeal carcinoma xenografts of C3H/He mice, and ferroptosis was also found to promote the increase of tumor-infiltrating CD4^+^ and CD8^+^ T cells. The results suggest that ferroptosis in HNSCC is closely related to the tumor immune response [77]. To investigate the role of ferroptosis in the prognosis and immunology of pan-cancer, key regulators of ferroptosis, solute vector family 7 member 11 (SLC7A11), glutathione peroxidase 4 (GPX4), and apoptosis-inducing factor mitochondria-associated 2 (AIFM2) were analyzed using TIMER, gene expression profile interaction analysis (GEPIA), Oncomine, and Kaplan–Meier databases. The expression level of AIFM2 in colorectal cancer, lung cancer, acute myeloid leukemia, esophageal cancer, and HNSCC was correlated with prognosis and invasion. Abnormal expression of SLC7A11, GPX4, and AIFM2 was found in a variety of tumors, which can be used to evaluate the prognosis and invasion degree of various cancers [78].

### 3.4. Ferroptosis and Drug Resistance

Traditional cytotoxic drugs and targeted drugs generally slow or stop tumor growth by inducing cancer cell death, leaving normal cells unaffected. However, the emergence of drug resistance during chemotherapy or targeted therapy remains a largely insurmountable challenge [16]. Studies have shown that ferroptosis is associated with drug resistance in cancer treatments, and inducing ferroptosis has been shown to reverse drug resistance [79,80]. Now, increasing preclinical evidence suggests that inducing ferroptosis may be an effective treatment strategy to prevent the acquired resistance to certain tumor therapies, such as gefitinib, lapatinib, erlotinib, trametinib, sorafenib, and vemurafenib [81,82]. Often some resistant cancer cells exhibit epithelial-mesenchymal transition, making them more sensitive to ferroptosis. Therefore, enhancing the sensitivity of drug-resistant cells to drugs by regulating ferroptosis is of great significance for improving the effectiveness of chemotherapy or targeted drugs in tumor therapy. Epithelial membrane protein (EMP1) overexpression has been reported to enhance RSL3-induced iron sagging in HNSCC cells by promoting gefitinib resistance through targeting the MAPK pathway [38]. Thus, the present research may implicate EMP1 as a promising therapeutic candidate for overcoming chemoresistance in HNSCC. Cisplatin is a commonly used chemotherapy agent in patients with unresectable locally advanced HNSCC, but its acquired resistance severely limits its use. Studies have shown that GlRX5-targeted short interfering RNA or short hairpin RNA (shRNA) can significantly promote the increase in lipid peroxidation and intracellular free iron in HNSCC cells, resulting in sulfasalazine- or cyst(e)ine-deprivation-induced ferroptosis. It was suggested that GLRX5 inhibition could induce ferroptosis in cisplatin-resistant HNSCC cells, thus enhancing the sensitivity of HNSCC cells to cisplatin [41]. Sulfasalazine induces ferroptotic cancer cell death by inhibiting the x_c_^−^-cystine/glutamate antiporter (xCT). An association was found between CISD2 expression and ferroptosis resistance. CISD2 overcomes HNC resistance to sulfazazine-induced ferroptosis cell death by increasing mitochondrial iron and lipid ROS accumulation [83].

## 4. Targeting Ferroptosis in the Prevention and Intervention of HNSCC

### 4.1. Ferroptosis and Cancer Diagnosis and Prognosis

Early diagnosis and prognostic monitoring hold immense importance in cancer treatment and prognosis. Evaluation of the correlation between ferroptosis-related genes (FRGs) and the prognosis of HNSCC will provide an important reference for our ability to predict recurrence of HNSCC patients. Therefore, researchers conducted a series of studies on the influence of FRGs on HNSCC prognosis through different research methods. Bioinformatics methods were used to study the correlation between FRGs and HNSCC prognosis, and it was found that ferroptosis-related lncRNA has predictive value in the prognosis evaluation of HNSCC [84]. Sun’s group analyzed the association of 11 FRGs with prognosis through the TCGA database, constructed a prognostic risk model, and further selected receiver operating characteristic (ROC), nomogram analysis, Gene Expression Omnibus, univariate cox regression analysis, and Kaplan–Meier curves. Finally, five ferroptosis-related molecular markers were selected that may affect the treatment targets of ferroptosis, providing new directions for the treatment of HNSCC [77]. Similar studies have shown that 10 differential FRGs in HNSCC were screened based on multiple public databases, and a prognostic risk model was constructed. Through analysis, the above genes were found to be closely related to HNSCC immune infiltration, immune checkpoint, and antitumor drug sensitivity. These results suggest that these genes may provide new potential therapeutic targets for individualized treatment of HNSCC patients [85]. In addition, bioinformatics methods have been used to analyze the role of immuno-ferroptosis-related mRNAs (IFRMs) in the treatment of HNSCC, and the prediction models of 12 IFRMs have been successfully constructed and grouped. Significant differences were found in survival prediction, immune regulation, and drug sensitivity in HNSCC patients. The construction of the IFRM tag will be beneficial to the prediction of the therapeutic effect of HNSCC [86].

### 4.2. Ferroptosis and HNSCC Therapeutic Strategy

Ferroptosis has garnered growing interest as a potential therapeutic approach for malignant tumors. With the rapid development of nanotechnology and biomaterial technology, multifunctional nano-mediated ferroptosis combination therapy has shown great promise for clinical application in tumor diagnosis and treatment [87,88,89]. Recently, research has shown that ferroptosis shows great potential in overcoming multi-drug resistance in cancer therapy [90]. DHA and sodium nitroprusside (SNP) are among the ferroptosis inducers. By loading SNP and DHA with surface-immobilized FA onto imidazoline-zeolite frame (Zif-8) nanoparticles, we synthesized DHA/SNP@Zif-8-FA nanocomposite drugs that target Fr-overexpressing HNSCC cells via ferroptosis. In vitro experiments showed that the nanoreactor could significantly increase intracellular ROS accumulation and reduce cell activity. In vivo experiments also showed that the nanoreactor could significantly inhibit the growth of tumors. In addition, indocyanine green was loaded into a nanoreactor DHA/SNP@Zif-8-FA (DHA/SNP/ICG@Zif-8-FA) to track and visualize the target. In conclusion, the nanoreactor may be a valuable therapeutic strategy for HNSCC [91]. In addition, the development and prognosis of HNSCC can also be influenced by some target genes related to ferroptosis. Ferroportin can inhibit the proliferation of HNSCC cells and arrest the cell cycle in the S phase. Further, ferroportin can reduce tumor growth in an oral orthotopic mouse xenotransplantation model [92]. These findings suggest that iron plays a role in HNSCC cell proliferation and sustained growth, and that treatment strategies based on ferroptosis may have potential therapeutic benefits. One study showed that overexpression of caveolin protein-1 (CAV1) in HNSCC tissues was associated with poor prognosis. It was also found that CAV1 could inhibit the ferroptosis signaling pathway of HNSCC. Ultimately, the researchers concluded that CAV1 could be a potential target for diagnosis and combination therapy strategies in patients with HNSCC [93]. 

### 4.3. Ferroptosis Resistance in HNSCC

Malignant tumors pose a serious threat to human health. Despite various clinical antitumor treatments, patients still have poor prognosis, frequent relapse, and metastasis, which eventually lead to death. How to overcome tumor treatment resistance remains an urgent clinical problem. Recent studies have shown that the ferroptosis pathway is closely related to tumor therapeutic efficacy. Although studies on ferroptosis have shown that it provides a new strategy for tumor therapy, cancer cells can acquire resistance to ferroptosis through upregulation of antiferroptosis proteins or downregulation of pro-ferroptosis proteins, thereby affecting the positive role of ferroptosis in tumor therapy [94]. This study focused on the regulatory role of interleukin-6 (IL-6) in the resistance to ferroptosis in HNSCC. It was found that the expression level of IL-6 gradually increased during the development of HNSCC, and the upregulation of xCT expression was associated with a poor prognosis of HNSCC. IL-6 not only activates xCT expression through JAK2/STAT3 signaling pathway transcription, but also reverses ferroptosis and growth inhibition induced by xCT knockout or ferroptosis inducer erastin. Therefore, the induction of ferroptosis resistance by IL-6 plays a significant role in the development of HNSCC [39].

## 5. Challenges and Prospects for Targeting Ferroptosis in Cancer Therapy

Ferroptosis, as a newly identified regulatory cell death mode, plays an important regulatory role in the occurrence and development of tumors. Although remarkable progress has been made in tumor biology and therapeutics, there is still a long way to go before cancer can be conquered. Given the potential of ferroptosis as a new therapeutic target, its role in tumor biology and therapy has been increasingly studied. However, there may be large gaps between cells, animals, and humans in this non-apoptotic mode of cell death, and further research is needed to bridge these gaps. It is worth noting that certain gene mutations have also been linked to ferroptosis. Research has demonstrated that cancer cells with mutated RAS are more susceptible to ferroptosis induction. There is evidence of synergistic effects between chemotherapeutic agents and ferroptosis-inducing agents in tumor treatment [95]. However, ferroptosis is a double-edged sword: it is necessary to fully study the potential toxic side effects of inducers or inhibitors of key proteins or pathways of ferroptosis, so as to ensure that the tumor specifically triggers the Fenton reaction and thus avoid causing cancer or other diseases in normal tissues [96]. A thorough understanding of the basic mechanisms underlying ferroptosis, including the pivotal roles of lipid peroxidation, iron metabolism, and glutathione pathways, is required. In addition, delving deeper into uncharted territory to identify essential molecules or pathways associated with ferroptosis holds the promise of uncovering novel targets for cancer therapy. For example, a recent discovery of FSP1, originally named AIFM2 due to it being homologous to the mitochondrial pro-apoptotic protein apoptosis inducer (AIFM1), serves as a glutathione-independent inhibitor of ferroptosis. Remarkably, pharmacological targeting of FSP1 in combination with GPX4 inhibitors shows a potent synergy in inducing ferroptosis in various cancer types [97,98]. 

In the future, the development of rational and safe antitumor drugs based on ferroptosis should be considered from many points of view. Given that only a low number of ferroptosis-associated drugs are available on the market, the discovery of small molecules capable of modulating key ferroptosis-related targets can be facilitated using high-throughput screening methods or virtual screening techniques. To improve the effectiveness of antitumor strategies based on ferroptosis, the exploration of potential synergies with other therapeutic modalities, such as immunotherapy or conventional chemotherapy, is warranted. Although evaluation of these potential synergies is imperative, it is critical to recognize that the specific strategies and targets of ferroptosis-based treatments may vary depending on the type of cancer being considered. Therefore, staying abreast of the latest research developments in this area is of paramount importance. In the interest of safety, extensive preclinical studies should be conducted to carefully evaluate the potential adverse effects of these drugs on normal cells and organs. Nevertheless, we believe that through deepening our basic research on ferroptosis, it will be possible to apply ferroptosis inducers or enhancers to sensitize tumors to therapy in the clinic. In conclusion, the research results surrounding the induction of ferroptosis will certainly bring new possibilities for cancer treatment.

## 6. Conclusions

With increasing research into cell death, it was not until the late 20th century that investigators realized that cell death was a regulated process. At the same time, ferroptosis, as a non-apoptotic form of cell death, is also considered a regulated process. The concept, characteristics, and related regulatory mechanisms of cell ferroptosis were discussed here to emphasize that it has important biological functions and physiological significance. To date, studies have preliminarily shown great prospects for the application of ferroptosis induction or inhibitors in the treatment of tumors. Additionally, the identification of target genes associated with ferroptosis has presented a novel therapeutic approach for diagnosing and prognosing tumors. Nevertheless, ferroptosis research into head and neck cancer is still in its early stages, highlighting the need for more in-depth and vigorous studies in this area.

## Figures and Tables

**Table 1:** Features, components, and detection methods of ferroptosis [23,24,25,26,27,28,29,30,31,32,33].

Ferroptosis	Features	Morphology: reduced mitochondrial volume, reduced or disappeared mitochondrial ridge, broken mitochondrial outer membrane, increased double membrane density
		Biochemical characteristics: Iron accumulation and lipid peroxidation
		Immune characteristics: DAMPs
		Key signature proteins: GPX4, p53, TFR1, SLC7A11, ACSL4, FSP1, Nrf2, Ferritin
	Detection methods	Morphological observations (transmission electron microscopy), determination of cell activity, iron levels, ROS and lipid peroxidation, glutathione levels, metabolomics or lipidomics, changes in expression levels of signature proteins.

**Table 2:** A summary of studies on ferroptosis in HNSCC.

Trigger	Targets	Regulation Mechanism	Ref.
EMT	GSH	EMT contributes to promoting ferroptosis	[36]
Ferroptosis stress	NF-κB	Ferroptotic stress induces the inflammation signature and PD-L1 expression	[37]
EMP1	Hippo-TAZ pathway, Rac1, and NOX1	EMP1 overexpression enhances RSL3-induced ferroptosis	[38]
IL-6	xCT	IL-6 transcriptionally upregulates xCT expression via the JAK2/STAT3 pathway	[39]
EREG	Lipid peroxidation, iron accumulation, and GPX4	EREG deficiency induces ferroptosis and enhances the sensitivity of HNSCC cells to cetuximab	[40]
Sulfasalazine and GLRX5	ROS and GSH	GLRX5 promotes ferroptosis by increasing the amount of intracellular free irons and lipid peroxidation	[41]
RSL3 and cetuximab	KRAS and FTH1	FTH1 reduces the susceptibility of HNSCC to ferroptosis inducers and prevents ferroptosis.	[42]
SOCS1 and FTH1	M1 and M2 macrophages	SOCS1 and FTH1 are independent prognostic factors that correlate with M1 and M2 macrophage infiltration in HNSCC	[43]
Artesunate	Nrf2-ARE pathway	Artesunate decreases cellular GSH level and increases lipid ROS level	[44]
Dihydroartemisinin (DHA)	ROS, GPX4, and Ras	DHA increases ROS levels and decreases GPX4 and Ras levels	[45]
HA15	HSPA5	HA15 reduces GPX4 and FTH1 expression and increases ACSL4 expression	[46]
RSL3 or ML-162	Nrf2-ARE pathway	RSL3 or ML-162 upregulates p62 and Nrf2 expression, downregulates Keap1 expression, and activates the PERK-ATF4-SESN2 pathway	[47]
Mitochondrial pyruvate carrier 1 (MCP1)	GPX4 and xCT	MPC1 increases the susceptibility to ferroptosis by regulating interstitial traits and glutaminolysis	[48]
Dihydrolipoamide dehydrogenase (DLD)	Cystine	DLD induces ferroptosis via cystine deprivation	[49]
	ACSL1, SLC39A14, TFRC, and PRNP	Unclear	[50]

## Abbreviation

AA	Arachidonoyl
ACD	Accidental cell death
ACSL4	Acyl-CoA synthetase 4
AdA	Adenoid/adrenoyl
CoA	Coenzyme A
DAMPs	Damage-associated molecular patterns
EMT	Epithelial mesenchymal transition
FRGs	Growth-related genes
GCL	Glutamate-cysteine ligase
GSH	Glutathione
GSS	Glutathione synthetase
GPX4	Glutathione peroxidase 4
HNSCC	Head and neck squamous cell carcinoma
ICB	Immune checkpoint blockade
IFN-γ	Interferon-γ
IFRMs	Immuno-ferroptosis-related mRNAs
LPCAT3	Lysophosphatidyllecithin acyltransferase 3
NADPH	Nicotinamide adenine dinucleotide phosphate oxidase
POR	P450 oxidoreductase
PUFA	Polyunsaturated fatty acid
ROC	Receiver operating characteristic
ROS	Reactive oxygen species
RCD	Regulated cell death
Tf	Transferrin
TFRC	Transferrin receptor
xCT	x_c_^−^-cystine/glutamate antiporter
